# Oral colchicine improves shoulder motion and reduces capsular fibrosis in a rat frozen shoulder model

**DOI:** 10.3389/ebm.2026.11233

**Published:** 2026-10-28

**Authors:** Gökay Eken, Adnan Kantarci, Teoman Atici, Ahmet Akkoc, Selcan Akesen

**Affiliations:** 1 Faculty of Medicine, Bursa Uludağ University, Bursa, Türkiye; 2 Bursa Uludag Universitesi Veteriner Fakultesi, Nilüfer, Türkiye

**Keywords:** adhesive capsulitis, antifibrosis, colchicine, frozen shoulder, rat model

## Abstract

Primary frozen shoulder is driven by capsular inflammation followed by progressive fibrosis. Current conservative treatments target inflammation alone and do not address the fibrosis that sustains the loss of motion. Since colchicine has both anti-inflammatory and antifibrotic activity, we tested whether oral colchicine limits inflammation, fibrosis, and the loss of shoulder motion in a rat immobilization model of frozen shoulder. Sixty female Wistar albino rats aged 8 months were randomly assigned to five groups of 12: a healthy sham group, a 3-week untreated control, a 3-week treatment group, a 6-week untreated control, and a 6-week treatment group. Frozen shoulder was induced by plaster immobilization in all groups except the sham group. The treated animals received 0.6 mg of oral colchicine daily by gavage, which is approximately equivalent to 2.2 mg/kg/day. Abduction and external rotation were measured radiographically on harvested specimens, and the joint capsule was graded histopathologically for synovial hyperplasia, inflammatory changes, pannus formation, capillary vessel formation, and connective tissue formation. At 6 weeks, the mean abduction angle was greater in the treatment group than in the matched control (109.6° versus 88.1°), as was the mean external rotation angle (26.8° versus 18.9°), whereas the 3-week groups did not differ significantly in abduction angles. Connective tissue formation was lower in both treatment groups than in their respective controls, and inflammatory changes were reduced at both time points. Immunohistochemistry showed that collagen III was lower in the 3-week treatment group than in its control (2.17 versus 3.50) and collagen I was lower in the 6-week treatment group than in its control (2.50 versus 3.50), a phase-dependent pattern. Differences in synovial hyperplasia, pannus formation, and capillary vessel formation did not reach significance. In this model, colchicine improved shoulder abduction and external rotation and reduced capsular fibrosis and inflammation. Colchicine, already used widely for rheumatologic disease, may have anti-inflammatory and antifibrotic potential in frozen shoulder, although further preclinical and clinical studies are needed before any clinical use. These findings concern the development and progression of capsular fibrosis during continued immobilization; whether colchicine can reverse established contracture was not tested.

## Impact statement

Frozen shoulder remains difficult to treat because the available conservative options reduce inflammation but leave untouched the capsular fibrosis that drives lasting stiffness. This study is the first to test colchicine, an inexpensive oral drug with a long clinical record in rheumatologic disease, against both processes in a controlled animal model of the condition. By timing treatment to the inflammatory and fibrotic phases separately, the work shows that colchicine can improve measurable joint motion while lowering capsular collagen deposition and inflammatory change in the same model. These findings identify a candidate worth evaluating further in adequately powered preclinical studies and, if those are supportive, in head-to-head comparison with corticosteroids. The results also support targeting capsular fibrosis directly, rather than inflammation alone, as a treatment strategy for adhesive capsulitis. The model addresses the prevention and attenuation of developing fibrosis rather than the treatment of established contracture, which is the setting in which patients usually present and which remains to be tested.

## Introduction

Primary frozen shoulder, also called adhesive capsulitis, is a painful condition in which both active and passive glenohumeral motion become progressively restricted. It usually begins spontaneously, without an identifiable trigger, and runs a long course that passes through overlapping inflammatory, fibrotic, and resolution phases, often described as the freezing, frozen, and thawing stages [1]. Its cause is still not fully understood, but evidence points to a self-perpetuating cycle in which synovial inflammation is followed by excessive capsular fibrosis. Proliferation of fibroblasts and myofibroblasts, new vessel formation, and dense collagen deposition thicken and contract the glenohumeral joint capsule [1, 2]. Because inflammation and progressive fibrosis are central to this process, they offer rational targets for drug treatment.

Current conservative treatment is primarily aimed at the inflammatory component. Nonsteroidal anti-inflammatory drugs and oral corticosteroids are widely used to relieve pain and inflammation. In a randomized controlled trial, a short, 3-week course of prednisolone at 30 mg/day gave significant short-term benefits, but corticosteroids carry adverse effects and are not recommended beyond 6 weeks [3]. None of these agents acts directly on the capsular fibrosis that underlies the lasting loss of motion. An agent that could reduce inflammation and suppress fibrosis at the same time would therefore meet an important unmet need.

Colchicine is a plant-derived alkaloid that has been used for more than 50 years to treat gout, familial Mediterranean fever, Behçet disease, scleroderma, amyloidosis, and hepatic cirrhosis, and it has both anti-inflammatory and antifibrotic properties [4, 5]. It binds to tubulin and inhibits microtubule polymerization, which suppresses neutrophil chemotaxis and inflammasome-driven cytokine release and interferes with fibroblast proliferation and collagen secretion. These actions map onto the inflammatory and fibrotic processes that occur in frozen shoulder [5]. In keeping with its antifibrotic activity, colchicine reduces post-inflammatory and post-surgical adhesion formation in several experimental models, including pelvic adhesions in rabbits [6], tunica albuginea fibrosis in rats [7], and peritoneal adhesions in rats [8]. Despite this rationale, the effect of colchicine on the inflammatory and fibrotic changes of frozen shoulder has not, to our knowledge, been studied.

Therefore, we hypothesized that systemic colchicine would reduce capsular inflammation and fibrosis and limit the loss of glenohumeral motion in an established rodent immobilization model of frozen shoulder [9, 10]. The aim of this study was to evaluate the effects of oral colchicine on the inflammatory and fibrotic stages of frozen shoulder, using radiographic measurements of joint motion together with histopathological assessments of the capsule.

## Materials and methods

### Ethics and animals

After approval from the local animal experimentation ethics committee (Decision no. 2021-03/08), 60 female Wistar albino rats, aged 8 months and weighing between 250 and 300 g were used. All animals were examined for anatomical abnormalities before inclusion. The rats were housed in pairs with *ad libitum* access to food and water at a room temperature of 20 ± 2 °C under a 12-h light/dark cycle.

### Sample size estimation

The primary outcome was the radiographically measured glenohumeral abduction angle at the end of treatment. The sample size was set *a priori* for a two-sided, independent samples comparison (α = 0.05, power = 0.80). Assuming a clinically meaningful between-group difference of 15° in abduction, with a standard deviation of 9° (Cohen’s d ≈ 1.7), based on previously reported rodent shoulder-contracture models [9, 10], six animals per outcome group were required. Twelve rats were therefore allocated to each of the five groups, with half (n = 6) used for radiographic range-of-motion (ROM) measurement and the other half (n = 6) for histopathological analysis. The histopathological parameters were predefined as secondary outcomes; therefore, the study was not specifically powered to detect differences in these semi-quantitative measures; this is acknowledged as a limitation.

### Randomization

Randomization was performed with the RANDBETWEEN function in Microsoft Excel. The 60 rats were divided into five groups of 12.

### Group S (healthy control)

This group served as the healthy control and confirmed that pair housing in a free cage with *ad libitum* food and water does not induce frozen shoulder. The ROM and histopathology of these animals served as reference data. At the end of the third week, the rats were euthanized, and their shoulder joints were harvested via scapulothoracic disarticulation.

### Group C1 (3-week untreated control)

A frozen shoulder model was created and left untreated. Shoulder ROM and histopathology represented the third-week disease state. The rats were euthanized at the end of the third week, and their shoulder joints were harvested via scapulothoracic disarticulation.

### Group T1 (3-week treatment)

A frozen shoulder model was created and treated with colchicine for its anti-inflammatory effects. Colchicine 0.6 mg/day was administered by gavage starting on the third day after immobilization, when inflammation and inflammatory cells were expected, and continued for 3 weeks. At the end of the third week, the rats were euthanized, and their shoulders were harvested via scapulothoracic disarticulation.

### Group C2 (6-week untreated control)

After the frozen shoulder model was applied, this group was left untreated. At 6 weeks, the rats were euthanized, and their shoulders were harvested via scapulothoracic disarticulation; shoulder ROM and histopathology represented the sixth-week disease state.

### Group T2 (6-week treatment)

A frozen shoulder model was created, and treatment focused on the antifibrotic effects of colchicine. Colchicine 0.6 mg/day was administered by gavage starting on the third week, when mature fibrosis was expected, and continued between the third and sixth week. These rats were euthanized at 6 weeks, and their shoulders were harvested via scapulothoracic disarticulation.

### Frozen shoulder model

In all groups except group S, frozen shoulder was induced by casting. Anesthesia was provided via sevoflurane (Sevorane 100%, AbbVie, Illinois, USA) delivered by inhalation at a concentration of 2–8%, and depth was confirmed by loss of the pedal reflex. The left forelimb was positioned as described by Kim et al. [9], with the shoulder in full adduction and internal rotation, and the elbow in flexion and pronation, and was immobilized with a cast applied around the thoracic wall (Figure 1). The casts were checked daily for integrity, loosening, and skin complications, and were replaced as needed; immobilization was maintained continuously until euthanasia (C1, and T1; 6 weeks for groups C2 and T2).

**FIGURE 1:**
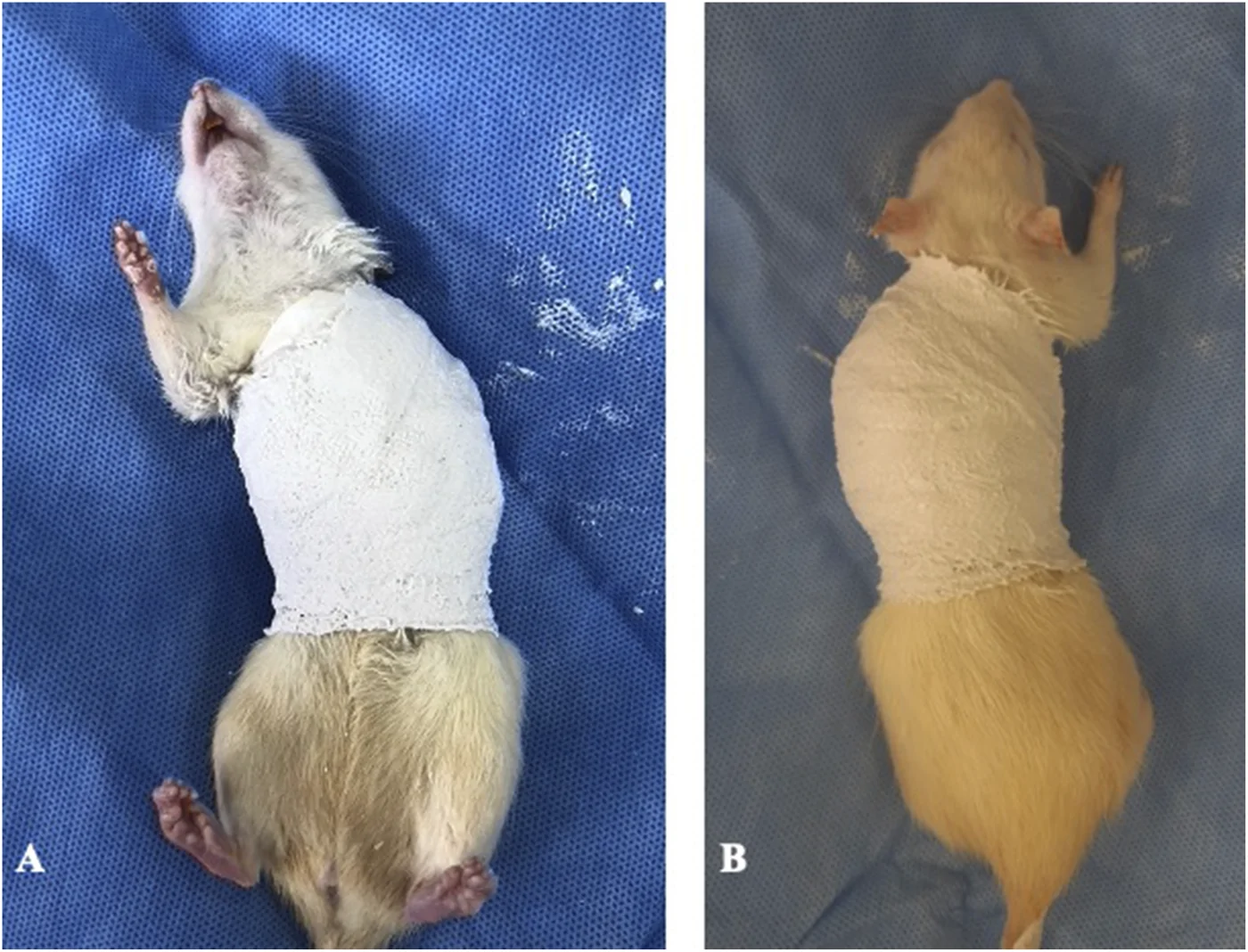
Positioning and plaster immobilization of the left forelimb used to induce the frozen shoulder model, with the shoulder in full adduction and internal rotation, the elbow in flexion and pronation, and the cast applied around the thoracic wall.

### Colchicine treatment and dosage

A rat dose of 0.5 ± 0.15 mg/day has been reported to approximate human therapeutic serum colchicine levels [11]. In this study, rats in groups T1 and T2 received 0.6 mg/day by gavage at a fixed time each day (09:00). For the 250–300 g body weight range, this corresponds to approximately 2.0–2.4 mg/kg/day (mean ≈2.2 mg/kg/day). The dose was therefore selected on a per-animal basis rather than a per-kilogram basis, following the regimen of the source study. Expressed per unit body weight, the dose is high relative to human therapeutic dosing, which reflects the faster clearance of colchicine in rats rather than an intention to exceed therapeutic exposure; serum colchicine concentrations were not measured in the present study. Two differences from the source protocol should also be noted: colchicine was administered as a once-daily gavage bolus rather than continuously in drinking water, which produces higher peak concentrations, and the daily dose was held constant rather than adjusted according to body weight during the treatment period. Animals were inspected daily when the casts were checked, and their general condition, stool consistency, and feeding behavior were noted at each inspection.

### Euthanasia and disarticulation

Rats in groups S, C1, and T1 were euthanized at the end of the third week, and those in groups C2 and T2 at the end of the sixth week, with a high dose of sevoflurane (Sevorane 100%, AbbVie, Illinois, USA). The cast was removed without moving the limb, and the forearm, humerus, and scapula were removed *en bloc*. During disarticulation, the shoulder joint was neither moved nor manipulated, since this could affect later ROM measurements (Figure 2).

**FIGURE 2:**
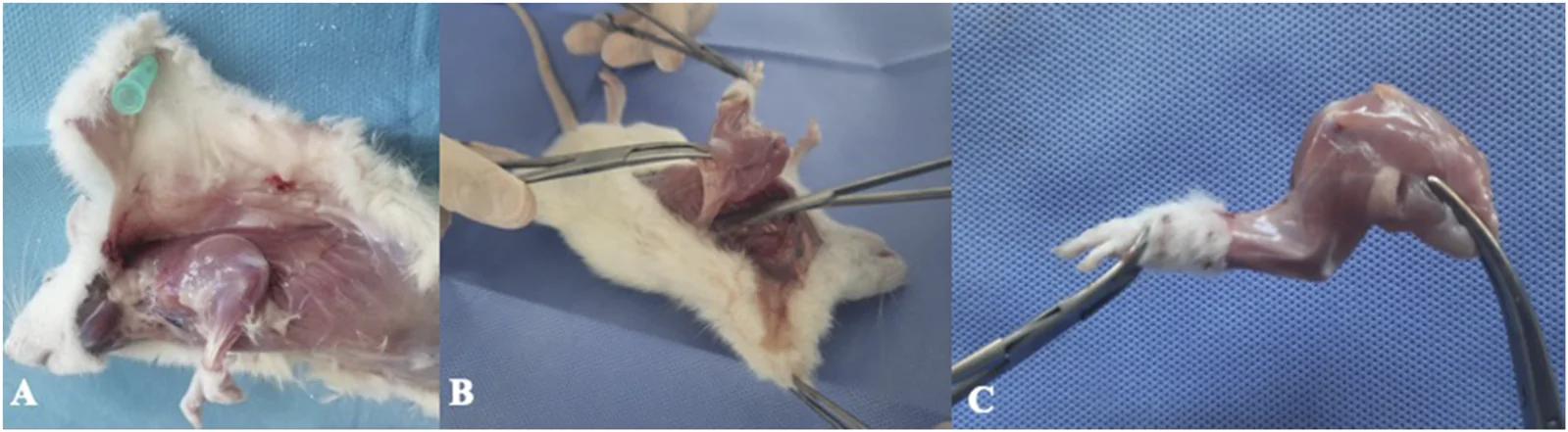
*En bloc* specimen (forearm, humerus, and scapula) harvested by scapulothoracic disarticulation without movement or manipulation of the shoulder joint.

### Radiographic ROM measurement (*ex vivo*)

ROM was assessed *ex vivo* on harvested specimens rather than live animals; therefore, the measurement reflects passive capsular stiffness rather than active, functional shoulder motion and is interpreted on that basis. Abduction and external rotation were measured using the method by Kanno et al. [10]. A standardized jig made of a foam board, a Bunsen support, and a clamp was used for stabilization. The scapula was fixed to the device, the center of the humeral head was aligned with the midpoint of the jig, and a 10-g torque was applied to the humeral shaft. After radiographic imaging, the two angles were measured using the four reference lines defined by Kanno et al. [10] (Figure 3). Radiographs were coded before measurement, and the angles were measured by an investigator blinded to group allocation.

**FIGURE 3:**
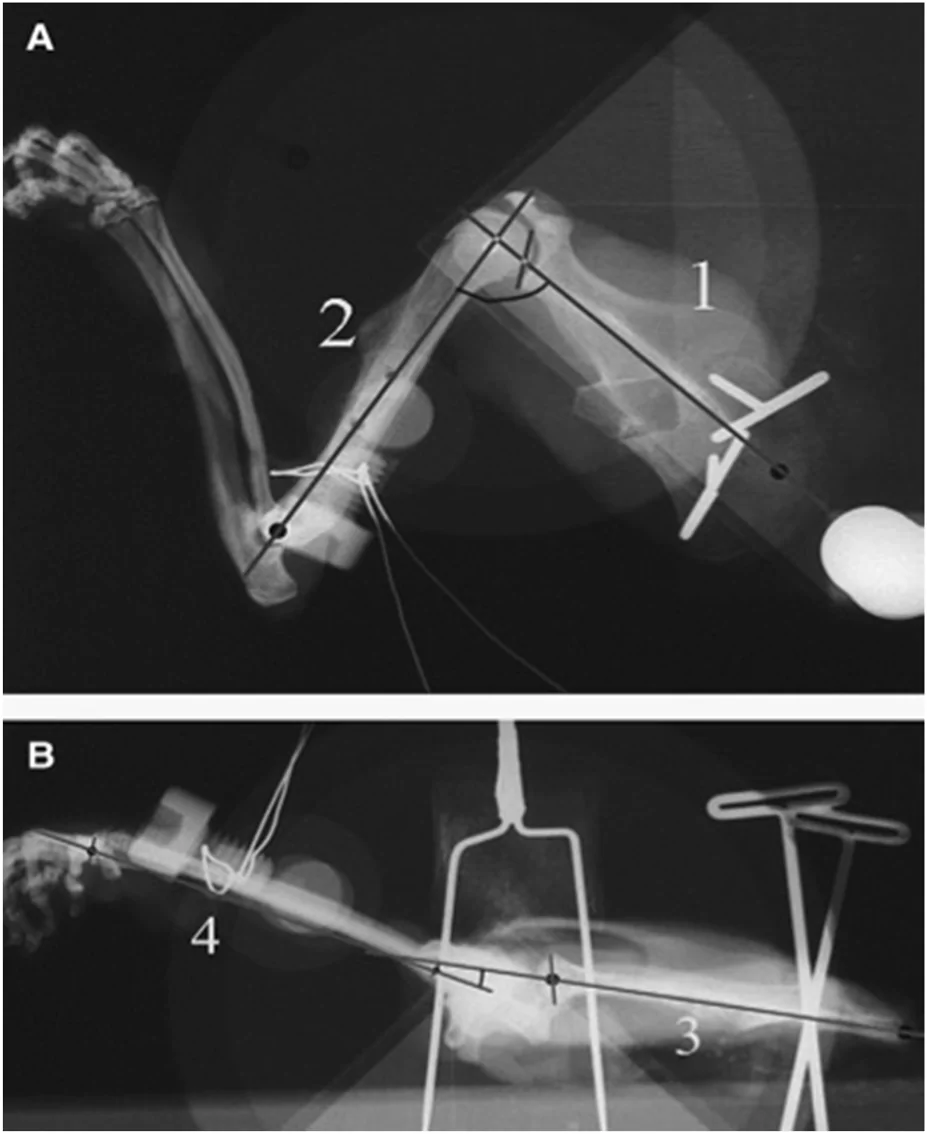
*Ex vivo* radiographic measurement setup. The scapula was fixed to a standardized jig, the center of the humeral head was aligned with the midpoint of the jig, and a 10 g torque was applied to the humeral shaft; abduction and external rotation were measured using the four reference lines.

### Histopathological evaluation

All specimens were fixed in 10% formaldehyde, coded, and evaluated by a pathologist blinded to group allocation. Synovial hyperplasia, inflammatory changes, pannus formation, capillary vessel formation, and connective tissue formation were graded semi-quantitatively on a 0-4 scale. Hematoxylin and eosin staining was used for synovial hyperplasia and inflammatory changes, and Masson’s trichrome staining was used for connective tissue formation, capillary vessel formation, and pannus formation. Glenohumeral joint sections were photographed under a ×4 objective after Masson’s trichrome staining, and pannus area was quantified in pixels with ImageJ (National Institutes of Health). Immunohistochemical staining was performed with a commercial kit (UltraVision Detection System Anti-Polyvalent HRP/DAB, TP-015-HD, Thermo Fisher Scientific, Waltham, MA, USA) to detect collagen I and collagen III in the glenohumeral joint region (Figure 4). The specimens were fixed for 48 h, after which they were decalcified in 14% EDTA (pH 7.2), with the solution renewed every 3 days. The glenohumeral joint was then cut anterolaterally toward the frontal plane perpendicular to the horizontal plane and embedded in paraffin. 4-µm sections were mounted on poly-L-lysine-coated slides (165,014, PatoLab, China) and held overnight at 37 °C. After deparaffinization and rehydration, antigen retrieval was performed in Tris-HCl in a microwave oven at 600 W for 2 × 5 min. The sections were washed in phosphate-buffered saline (4 × 5 min), treated with hydrogen peroxide for 15 min to block endogenous peroxidase, and incubated with protein block for 5 min. Primary antibodies were applied overnight at 4 °C in a humidified chamber: collagen I monoclonal antibody (bsm-33400M, Bioss Antibodies Inc., Massachusetts, USA) and collagen III polyclonal antibody (bs-0549R, 1:100, Bioss Antibodies Inc., Massachusetts, USA). An HRP-conjugated secondary antibody was then applied for 30 min in the dark, followed by DAB Plus chromogen for 3 min. The sections were counterstained with Harris hematoxylin (HHS16, Sigma-Aldrich) for 45 s, cleared in xylene, and mounted. A histopathological and immunohistochemical assessment was performed on the synovium of the glenohumeral joint and the subsynovial tissue of the axillary recess. Collagen I and collagen III immunostaining were graded on the same 0-4 semi-quantitative scale by the same pathologist, who remained blinded to group allocation. The following details were not reported: the working dilution of the collagen I antibody (which the source does not state), the positive and negative controls used, the objective magnification, and the number of fields assessed per animal. Densitometric image analysis was not performed for the collagen stains. Photomicrographs were archived for representative sections rather than systematically for every animal and field, and were not acquired under the standardized illumination and exposure settings that quantitative densitometry requires; thus, a valid measurement of positive area across all groups could not be obtained retrospectively. Collagen I and collagen III staining and scoring were carried out on sections from the same specimens used for the other histopathological parameters, harvested at 3 and 6 weeks. The sections were taken from the synovium of the glenohumeral joint and the subsynovial tissue of the axillary recess, cut from the anterolateral to the frontal plane, perpendicular to the horizontal plane, and this region was assessed in every animal. Whole-slide digital scanning was not performed.

**FIGURE 4:**
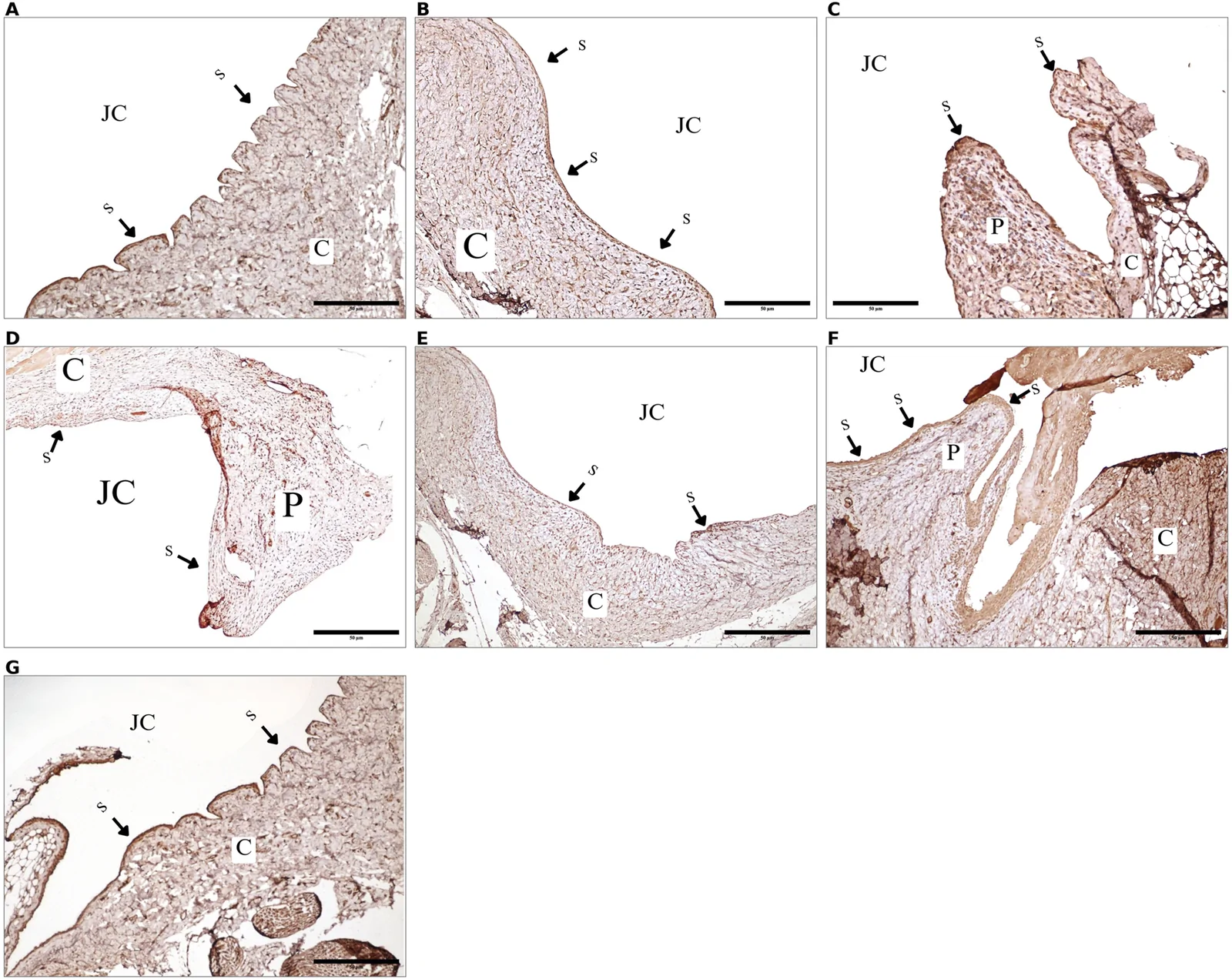
Collagen I and collagen III immunohistochemistry of the glenohumeral joint capsule. Panels **(A–C)** show collagen I staining graded 0 (A, Bar = 50 μm, S: synovial membrane, C: capsular tissue, JC: joint cavity), 2 (B, Bar = 50 μm, S: synovial membrane, C: capsular tissue, JC: joint cavity), and 3 (C, Bar = 50 μm, S: synovial membrane, C: capsular tissue, JC: joint cavity); panels **(D–F)** show collagen III staining graded 0 (D, Bar = 50 μm, S: synovial membrane, C: capsular tissue, JC: joint cavity, P: pannus tissue), 2 (E, Bar = 50 μm, S: synovial membrane, C: capsular tissue, JC: joint cavity, P: pannus tissue), and 3 (F, Bar = 50 μm, S: synovial membrane, C: capsular tissue, JC: joint cavity). These panels illustrate the semi-quantitative grading scale applied to all sections. Panel **(G)** shows a representative collagen III section from group T2, the delayed-treatment group (Bar = 50 μm, S: synovial membrane, C: capsular tissue, JC: joint cavity, P: pannus tissue). Scoring of collagen I and collagen III was performed on all animals in the histopathology subgroup (n = 6 per group); the panels shown here are illustrative and do not show one section per group.

### Statistical analysis

Categorical variables were summarized as counts and percentages, and continuous variables were summarized as medians (interquartile ranges). Because the data did not meet parametric assumptions, between-group comparisons of continuous variables used the Kruskal-Wallis H test and the Mann-Whitney U test. Post hoc analyses used the Dunn-Bonferroni correction to account for multiple comparisons. Categorical variables were compared with the chi-square test or the Fisher exact test. A two-sided p < 0.05 was considered statistically significant. Analyses were performed with IBM SPSS Statistics 28.0 (IBM Corp., Armonk, NY, USA). For collagen I and collagen III, two treatment-versus-control contrasts were specified *a priori* for each marker, corresponding to the two treatment arms of the study design: group T1 versus group C1 at 3 weeks and group T2 versus group C2 at 6 weeks. These are the contrasts for which the study was statistically powered, and the Bonferroni correction was applied within each marker across these two planned comparisons. All other between-group comparisons were exploratory and were corrected across all pairwise comparisons with the Dunn-Bonferroni procedure. Effect sizes with 95% confidence intervals are reported alongside p-values for the two pre-specified treatment-versus-control contrasts (Table 1). Hedges’ g is reported for the continuous outcomes and Cliff’s delta for ordinal collagen scores; both are expressed as treatment minus control; thus, a negative value indicates a lower score in the colchicine-treated group.

**TABLE 1:** Effect sizes with 95% confidence intervals for the two pre-specified treatment-versus-control contrasts.

Outcome	3 weeks: T1 vs. C1	6 weeks: T2 vs. C2
Abduction (°)	g = +0.12 (−1.01 to +1.26)	g = +2.32 (+0.80 to +3.84)
External rotation (°)	g = +0.29 (−0.85 to +1.42)	g = +0.62 (−0.55 to +1.78)
Connective tissue formation	g = −0.44 (−1.58 to +0.71)	g = −0.15 (−1.28 to +0.98)
Synovial hyperplasia	g = −1.15 (−2.39 to +0.09)	g = −0.25 (−1.39 to +0.88)
Inflammatory changes	g = −0.62 (−1.78 to +0.55)	g = −0.42 (−1.56 to +0.73)
Capillary vessel formation	g = 0.00 (−1.13 to +1.13)	g = −0.42 (−1.56 to +0.73)
Pannus formation (pixel area)	g = −0.08 (−1.21 to +1.05)	g = −0.20 (−1.33 to +0.94)
Collagen I	δ = −0.42 (−0.94 to +0.22)	δ = −0.75 (−1.00 to −0.44)
Collagen III	δ = −0.83 (−1.00 to −0.56)	δ = −0.25 (−0.78 to +0.36)

Effect sizes are expressed as treatment minus control, so a negative value indicates a lower score in the colchicine-treated group. Hedges’ g is reported where only group means and standard deviations were available; Cliff’s delta (δ) is reported for collagen I and collagen III, for which the individual animal scores were available and which are ordinal. A confidence interval that includes zero is compatible with no difference.

## Results

### Tolerability

All 60 animals completed the study and were included in the analysis; no animal died prematurely, and none was withdrawn. No diarrhea or other gastrointestinal signs were observed in any group, and no gavage-related complications occurred. Body weight was recorded at baseline but was not monitored serially during treatment.

### Radiographic outcomes

The abduction and external rotation angles are listed in Table 2, and their distribution across the groups is shown in Figure 5. The mean abduction angle was 88.1° in group C2 and 109.6° in group T2, a mean improvement of 21.5° that was statistically significant (p = 0.002). (Hedges’ g = 2.32, 95% CI 0.80–3.84). The mean abduction angle in groups C1 and C2 was significantly smaller than in group S (p = 0.006 and p < 0.001, respectively). Abduction in group T1 was also significantly smaller than in group S (p = 0.026), whereas group T2 and group S did not differ significantly (p = 0.718). External rotation followed a similar pattern. The mean external rotation was 18.9° in group C2 and 26.8° in group T2, a mean improvement of 7.9° that was statistically significant (p = 0.005). (Hedges’ g = 0.62, 95% CI 0.55–1.78). External rotation was significantly higher in group T2 than in group C1 (p < 0.001) and was significantly smaller in groups C1 and C2 than in group S (p = 0.003 and p = 0.013, respectively).

**TABLE 2:** Results of abduction and external rotation for all groups.

Variable	Group	N	Mean	SD	Minimum	Maximum
Abduction (°)	Group S	6	112.41	12.01	98.66	132.00
​	Group C1	6	94.41	4.12	86.30	97.56
​	Group T1	6	95.32	8.69	79.68	104.50
​	Group C2	6	88.07	7.97	75.73	98.61
​	Group T2	6	109.60	9.13	95.10	120.60
External rotation (°)	Group S	6	29.22	10.03	13.87	42.54
​	Group C1	6	18.12	7.05	5.64	26.76
​	Group T1	6	20.22	6.53	10.39	28.19
​	Group C2	6	18.88	9.87	6.74	32.78
​	Group T2	6	26.79	13.53	14.40	47.06

Abbreviations: N, number; SD, standard deviation. Angles are expressed in degrees (°).

**FIGURE 5:**
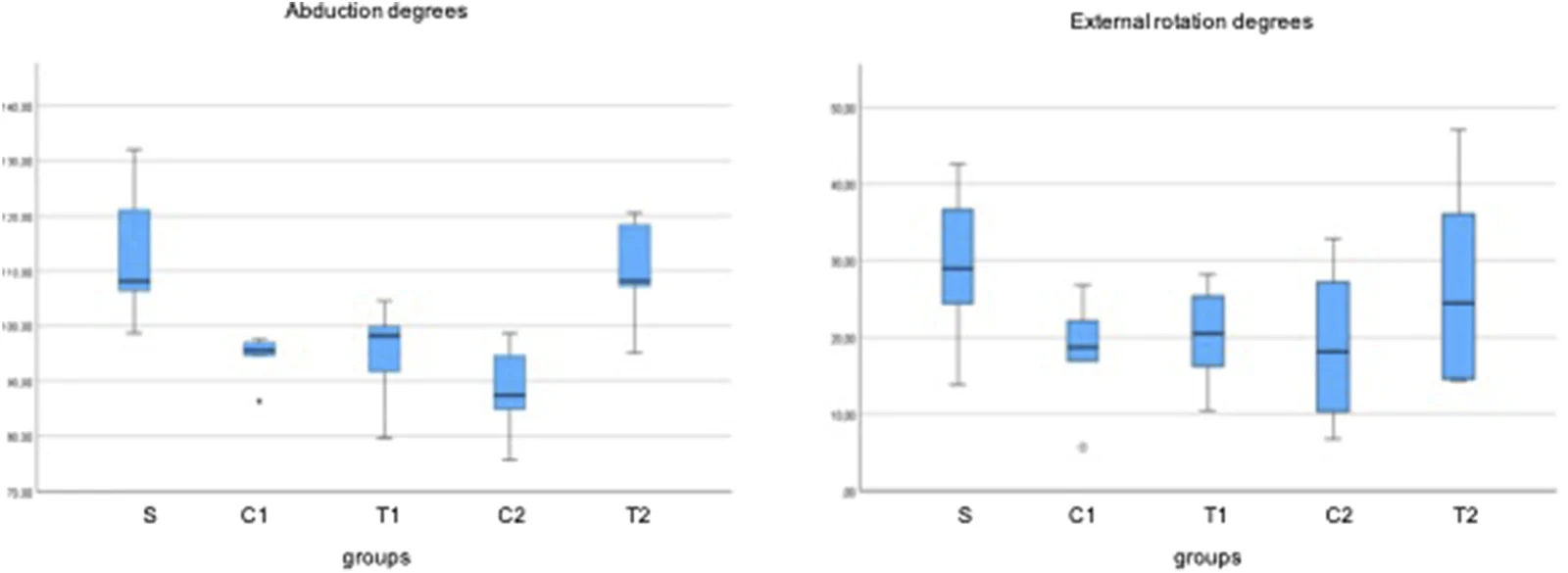
Distribution of abduction and external rotation angles across the five study groups. n = 6 per group.

All histopathological parameters and group means are listed in Table 3.

**TABLE 3:** Histopathological outcomes of all groups.

Parameter	Group S		Group C1		Group T1		Group C2		Group T2	
​	Mean	SD	Mean	SD	Mean	SD	Mean	SD	Mean	SD
Connective tissue formation	0.50	0.84	1.83	1.72	1.17	0.98	1.67	0.52	1.50	1.38
Synovial hyperplasia	0.00	0.00	1.33	1.51	0.00	0.00	0.83	1.17	0.50	1.22
Inflammatory changes	0.00	0.00	2.50	1.76	1.33	1.75	1.83	0.98	1.17	1.83
Capillary vessel formation	1.00	0.89	2.83	0.75	2.83	0.41	2.17	0.75	1.83	0.75
Pannus formation (pixel area)	0.00	0.00	375,394	266,752	348,620	364,695	588,772	250,931	533,957	255,057
Collagen I	0.17	0.41	2.17	0.98	1.50	0.55	3.50	0.55	2.50	0.55
Collagen III	0.33	0.52	3.50	0.55	2.17	0.75	2.17	0.75	1.83	0.75

Abbreviations: SD, standard deviation. Connective tissue formation, synovial hyperplasia, inflammatory changes, and capillary vessel formation were graded semi-quantitatively on a 0–4 scale; pannus formation is expressed as pixel area. Collagen I and collagen III were graded on the same 0–4 semi-quantitative scale by the same blinded pathologist.

Effect sizes with 95% confidence intervals for the two pre-specified treatment-versus-control contrasts are given in Table 1.

### Connective tissue formation

The mean values in groups T1 and T2 were significantly lower than in groups C1 and C2 (p = 0.012 and p = 0.031, respectively), indicating a significant reduction in capsular connective tissue formation in the colchicine-treated groups.

### Synovial hyperplasia

The mean values in groups T1 and T2 were lower than in groups C1 and C2, but neither difference was significant (p = 0.097 and p = 0.081, respectively).

### Inflammatory changes

Inflammatory changes were graded from 0 to 4 according to severity, and the mean scores were compared between the groups (Figure 6). The mean score in group S was significantly lower than in groups C1 and C2 (p = 0.003 and p = 0.011, respectively). The mean score decreased from 2.50 in group C1 to 1.33 in group T1, and from 1.83 in group C2 to 1.17 in group T2; both reductions were significant (p = 0.020 and p = 0.032, respectively).

**FIGURE 6:**
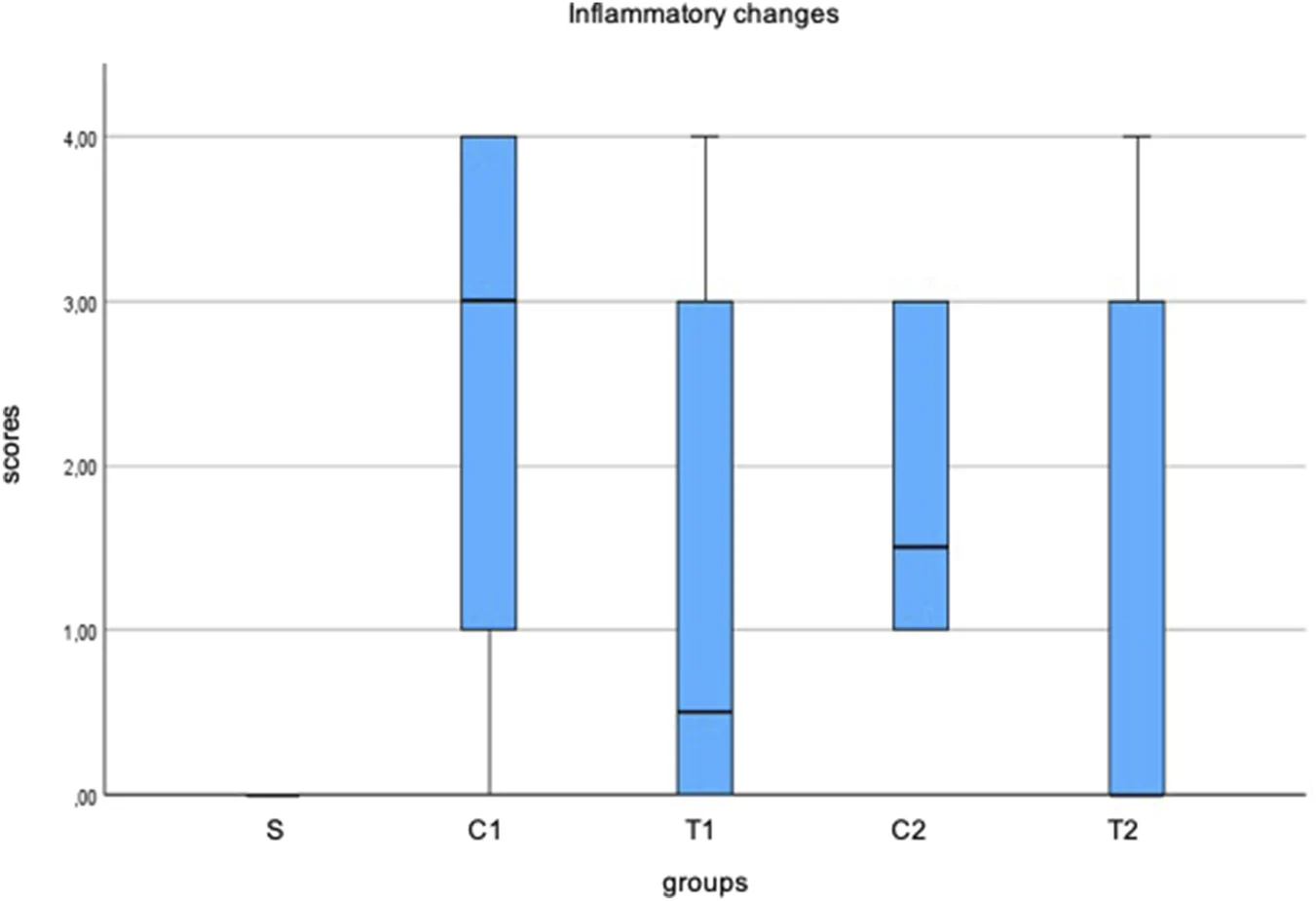
Mean inflammatory change scores (graded 0–4) across the five study groups. n = 6 per group.

### Capillary vessel formation

New capillary vessel formation was graded from 0 to 4 according to severity, and the mean scores were compared between the groups (Figure 7). The mean score in group S (1.00) was significantly lower than in groups C1 and T1 (both 2.83; p = 0.002 and p = 0.001). The mean score was significantly lower in group T2 (1.83) than in group T1 (2.83) (p = 0.038). The remaining comparisons were not significant (p > 0.05).

**FIGURE 7:**
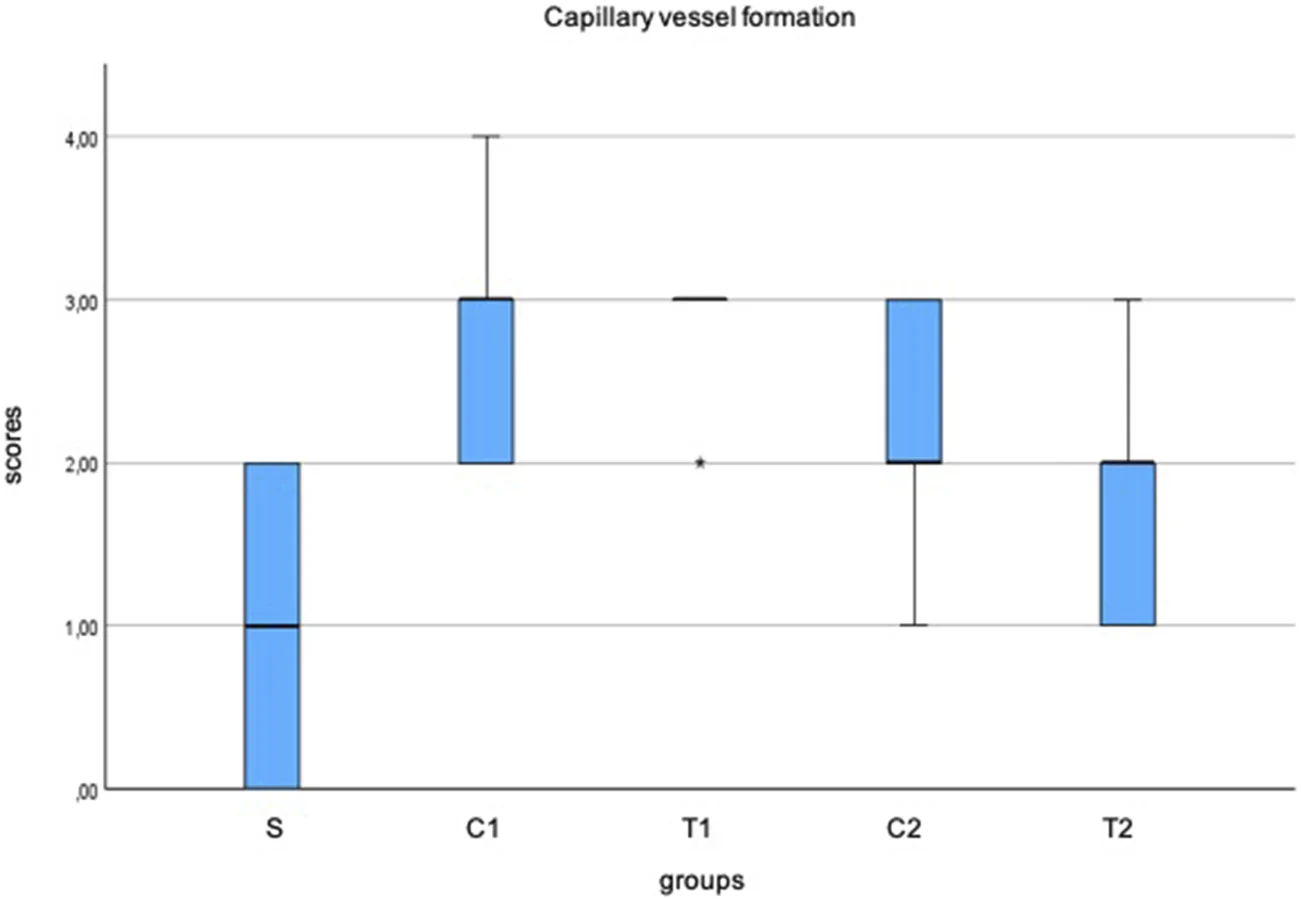
Mean new capillary vessel formation scores (graded 0–4) across the five study groups. n = 6 per group.

### Pannus formation

Pannus formation was quantified as pixel area, and mean values were compared between the groups (Figure 8). The mean pannus formation in groups C1, T1, C2, and T2 was significantly higher than in group S (p = 0.014, 0.019, 0.001, and 0.001, respectively). Pannus formation was lower in group T1 than in group C1 and in group T2 than in group C2, but these differences were not significant (p = 0.895 and p = 0.792, respectively).

**FIGURE 8:**
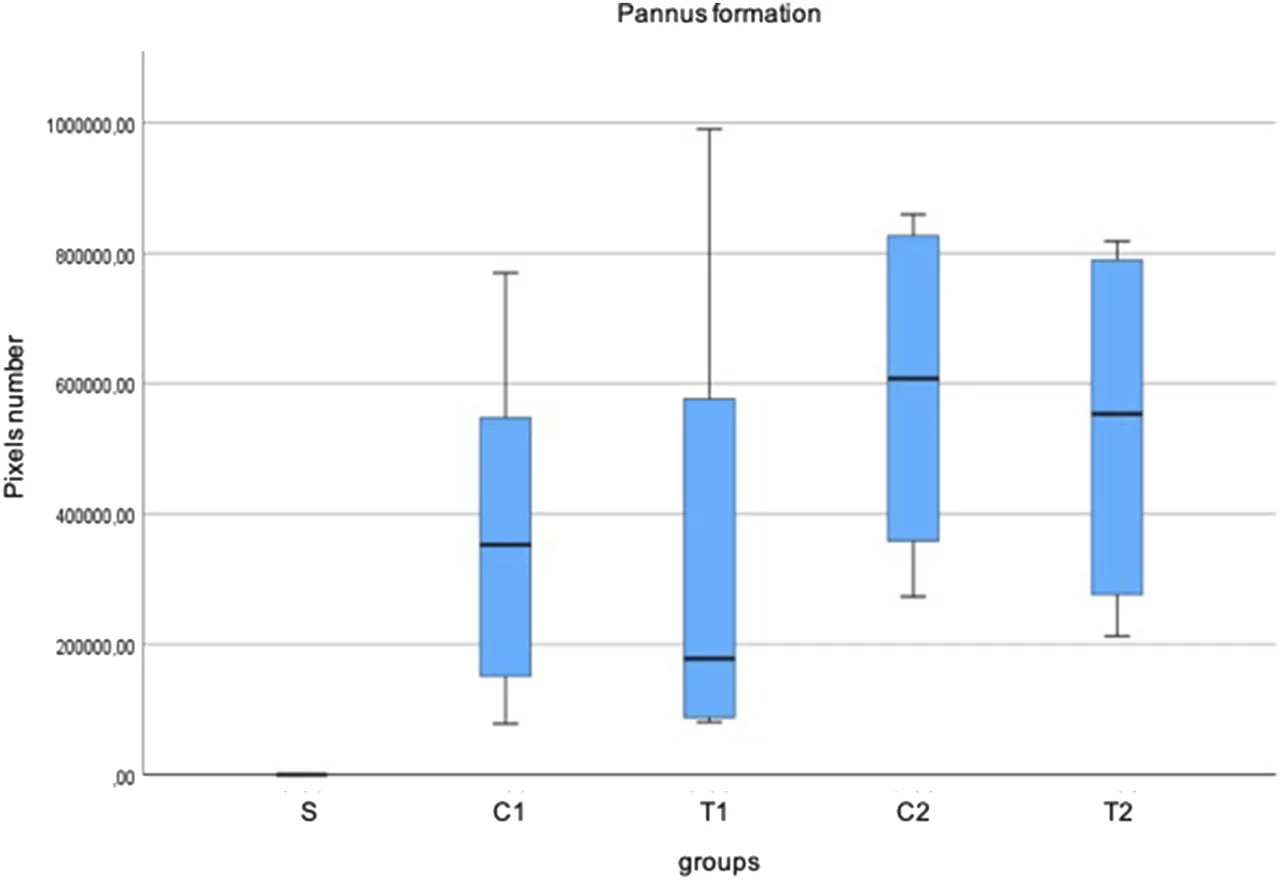
Mean pannus formation (pixel area) across the five study groups. n = 6 per group.

### Collagen I and collagen III expression

The collagen I and collagen III scores are listed in Table 3, and representative stained sections are shown in Figure 4. In the untreated groups, collagen I increased with the duration of immobilization (with a mean score of 2.17 in group C1 and 3.50 in group C2), whereas collagen III was highest at 3 weeks and lower at 6 weeks (3.50 and 2.17), a pattern consistent with the maturation of the capsular matrix from immature collagen III toward collagen I. The two pre-specified treatment-versus-control contrasts showed a phase-dependent effect. At 3 weeks, collagen III was lower in group T1 than in group C1 (2.17 versus 3.50, p = 0.029) (Cliff’s δ = −0.83, 95% CI −1.00 to −0.56), whereas collagen I did not differ significantly (1.50 versus 2.17, p = 0.465). At 6 weeks, the pattern was reversed: collagen I was lower in group T2 than in group C2 (2.50 versus 3.50, p = 0.048) (Cliff’s δ = −0.75, 95% CI −1.00 to −0.44), whereas collagen III did not differ significantly (1.83 versus 2.17, p = 0.974). Across all five groups, the overall difference was significant for both markers (Kruskal-Wallis p < 0.001 for each). Following Dunn-Bonferroni correction, exploratory comparisons with group S revealed that collagen I was higher in groups C2 and T2 (p < 0.001 and p = 0.023, respectively) and collagen III was higher in group C1 (p < 0.001); the remaining comparisons with group S did not reach significance after correction.

## Discussion

In this model, colchicine improved both the functional and the histopathological indices of the induced contracture. Radiographic joint motion in the treated rats was significantly better, with greater abduction (p = 0.002) and external rotation (p = 0.005) in the 6-week treatment group than in its matched control. These functional gains were accompanied by matching histopathological changes: connective tissue formation was significantly lower in the treatment groups than in their controls at both 3 and 6 weeks (p = 0.012 and p = 0.031, respectively), and inflammatory changes were also significantly reduced (p = 0.020 and p = 0.032, respectively). The sham group outperformed the control groups both radiographically and histopathologically, which confirms that the immobilization model reliably produced frozen shoulder. The agreement between the functional and histological findings supports an anti-inflammatory and antifibrotic effect of colchicine in this model. Immunohistochemistry for collagen I and collagen III, reported here for the first time, followed the same direction: treatment lowered collagen III during the inflammatory phase and collagen I during the fibrotic phase.

Although rats are quadrupeds, they stand on their hind legs to feed and use their forelimbs fairly freely, which means that they reproduce human shoulder movement better than dogs or rabbits. Soslowsky et al. [12] reported that rat shoulder motion during forward locomotion corresponds to human abduction. In human frozen shoulder, the greatest loss of motion occurs in external rotation, whereas in the rat model it is in abduction [9], probably because rats use the abduction arc more in daily activity. In line with this, the clearest improvement in our treatment groups was also observed in abduction.

ROM here was measured *ex vivo* on harvested specimens under a standardized 10-g torque [10] immediately after cast removal and without manipulating the joint. This reduces confounding factors arising from daily *in vivo* loading and provides reproducible measurements; however, it reflects passive capsular stiffness rather than active, functional shoulder motion, and the findings should be read accordingly. The parallel and statistically significant reduction in capsular connective tissue formation seen histologically is consistent with a real decrease in capsular contracture rather than a measurement artifact.

Static angular measurement under a fixed dead-weight torque also describes only one point in the range. Glenohumeral motion *in vivo* is multi-axial and is accompanied by joint reaction forces that vary through the arc, and capsular contracture not only changes the end-range angle, but also the resistance encountered across the whole arc. Instrumented testing would address this directly by driving the joint through a defined arc while a torque sensor continuously records resistance to yield torque-angle curves. These curves can be used to derive joint stiffness, the width of the neutral zone, and hysteresis, meaning that the response to treatment can be described as a change in the shape of the curve rather than as a change in a single angle. Such testing can be performed *ex vivo* on harvested specimens to preserve the reproducibility of the present approach, or *in vivo* under anesthesia during continuous passive motion to additionally capture the contribution of the periarticular muscles. We have incorporated instrumented torque-angle measurement into the design of our planned comparison of colchicine with corticosteroids in this model.

Frozen shoulder animal models were first developed by Schollmeier et al. [13], who immobilized canine shoulders with casts; this resulted in reduced motion, increased intra-articular pressure, synovial hyperplasia, and increased vascularity and adhesions in the subacromial bursa, with recovery once motion resumed. Rat shoulder immobilization models using internal fixation have also been described [10, 14], but although internal fixation may seem reliable, dissection and hardware placement can cause additional iatrogenic intra-articular damage. We therefore chose a plaster immobilization model, which is simple, easy to apply, and effective with minimal side effects. Liu et al. [2] applied a cast to the rat shoulder, but did not measure ROM and used a follow-up of only 4 weeks, after which they concluded that their model was unsuitable as a frozen shoulder model; nevertheless, their histological observations are valuable, showing that the anterior capsular synovial hyperplasia present at 2 and 3 weeks had resolved by the fourth week and confirming that frozen shoulder begins with an inflammatory phase. Consistent with the literature, synovial hyperplasia in our study was prominent at 3 weeks and had decreased by 6 weeks.

Kim et al. [9] observed inflammation by the third day and mature fibrosis from the third week onward in their model. We built our two treatment arms around these two phases to investigate the two main mechanisms of colchicine. In group T1, colchicine treatment began on the third day after casting to target the inflammatory phase; in group T2, it began at 3 weeks to target the established fibrotic phase. Colchicine binds to tubulin and inhibits microtubule polymerization, which suppresses neutrophil chemotaxis and inflammasome-mediated cytokine release, in addition to interfering with fibroblast proliferation and collagen deposition [5]. These mechanisms are consistent with our findings, although we did not test them directly: inflammatory changes were significantly reduced in the early treatment group, and connective tissue formation was significantly reduced in both treatment groups, which suggests that colchicine was active across both the inflammatory and the fibrotic phases. Pannus formation and synovial hyperplasia were lower in the treatment groups, but did not reach significance, and new capillary vessel formation differed significantly only between the two treatment groups (T1 versus T2, p = 0.038), indicating that the anti-angiogenic and anti-proliferative effects were less consistent than the anti-fibrotic and anti-inflammatory effects.

The collagen I and collagen III data directly support this phase specificity. In the untreated groups, the capsular matrix shifted from collagen III toward collagen I between weeks 3 and 6, which is the expected course of maturing fibrosis. Colchicine lowered whichever collagen type predominated during the period in which it was administered: collagen III in group T1, which was treated from the third day through the inflammatory phase, and collagen I in group T2, which was treated from the third week through the fibrotic phase. The correspondence between the timing of treatment and the affected collagen type is consistent with an effect on matrix deposition in both phases. It does not identify the pathway involved. With six animals contributing to each endpoint, statistical significance carries limited information about biological magnitude, so the effect sizes and confidence intervals in Table 1 should be read alongside the p-values. The intervals are wide throughout, and several are compatible with no difference; the abduction gain at 6 weeks is the only effect estimated with reasonable precision.

A distinction should be drawn between preventing fibrosis and reversing it. In group T1, colchicine was started on the third day after immobilization, before mature fibrosis had developed; therefore, the effect in that arm is one of prevention. In group T2, treatment began at 3 weeks, when mature fibrosis is present in this model; however, immobilization was maintained until euthanasia at 6 weeks. The comparison with group C2 therefore measures attenuation of continuing fibrosis under a continuing stimulus, rather than resolution of fibrosis after the stimulus has been removed. Neither arm tested whether colchicine can reverse established capsular contracture, and the present design cannot answer this question, since each animal was measured once, at the time of euthanasia, and no within-animal change from a pre-treatment state can be demonstrated. Patients with adhesive capsulitis usually present after capsular fibrosis is established, which is the setting in which colchicine would have to work. Testing it requires a model in which immobilization is discontinued once fibrosis has developed, treatment is then started, and motion is compared with that of untreated animals allowed to remodel spontaneously. We regard such a study as a necessary step before clinical translation, and the present findings should be read as evidence that colchicine limits the development and progression of capsular fibrosis, rather than as evidence that it treats established disease.

Several considerations limit what this model can say about clinical use. The rat shoulder differs from the human shoulder in its loading and in its arc of motion, and the distribution of motion loss also differs, with abduction being most affected in rats and external rotation being most affected in humans. The model also produces contracture through external immobilization rather than by the spontaneous synovial inflammation that defines primary frozen shoulder. Colchicine is handled differently across species: clearance is faster in rats, which means that the weight-normalized dose used here is not equivalent to a therapeutic dose in humans, and we did not measure serum concentrations to establish what exposure was achieved. In humans, colchicine has a narrow therapeutic index, variable oral bioavailability, and clinically important interactions via CYP3A4 and P-glycoprotein, with renal and hepatic impairment further affecting clearance; none of this variability is represented by a single fixed dose administered to healthy young animals over a few weeks. The study also included no active comparator, meaning that the effects reported here cannot be weighed against those of a short course of corticosteroids, which is an established treatment. Taken together, these considerations place the present work as an exploratory preclinical study that identifies a hypothesis worth testing rather than as evidence that colchicine is an effective conservative treatment option for frozen shoulder.

This study has several limitations. The sample size was modest and, because the histopathological parameters were predefined as secondary outcomes, the study may have been underpowered to detect smaller differences in these measures; the non-significant trends in pannus formation and synovial hyperplasia should therefore be read with caution, and larger studies are warranted. As noted, ROM was assessed *ex vivo* and reflects passive capsular stiffness rather than functional motion. Only one species and one sex were studied. Because rat fur is soft and slippery and immobilized limbs lose muscle bulk over time, minor movement within the cast cannot be entirely excluded; to our knowledge, external and internal fixation have not been compared directly, which merits future study. Finally, although a short course of corticosteroids is an established treatment for frozen shoulder [3], colchicine has not been studied in this condition before and was not compared here with an active comparator; a head-to-head comparison of colchicine and corticosteroids is planned. Upstream profibrotic mediators, including TGF-β1, α-SMA, CTGF, and fibronectin, were not measured. The present data therefore demonstrate a reduction in capsular collagen deposition rather than inhibition of profibrotic signaling, and the mechanism by which colchicine produces this effect remains to be established. The collagen findings rest on two planned comparisons in six animals per group and should be regarded as preliminary. α-SMA and TGF-β1 immunohistochemistry have been incorporated into the design of the planned comparison. Tolerability was assessed by daily clinical inspection alone: body weight was not monitored serially during treatment, and serum colchicine concentrations were not measured; therefore, we cannot confirm that systemic exposure corresponded to the human therapeutic range. A single dose level was studied, so no dose-response relationship could be inferred. The study design also tested prevention and attenuation of developing fibrosis rather than treatment of established contracture, since immobilization continued throughout the treatment period in both treatment arms. Collagen I and collagen III were assessed by semi-quantitative scoring rather than using quantitative image analysis, which limits the resolution of these measurements; standardized digital image acquisition and densitometric analysis have been incorporated into the design of our planned follow-up study. Histopathological scoring was performed on selected fields within a single capsular region rather than across whole-slide sections; therefore, it does not characterize the structural integrity of the joint as a whole, and regions such as the subacromial bursa were not assessed separately. Whole-slide imaging with regional mapping has been incorporated into the design of our planned follow-up study. Archived photomicrographs were captured to illustrate the grades of the scoring scale rather than one section per experimental group, meaning that the figures cannot show matched high-power fields for every group. Markers of myofibroblast differentiation (α-SMA) and of inflammatory signaling (COX-2 and IL-1β) were not assessed either, so the anti-inflammatory and antifibrotic effects reported here are inferred from tissue outcomes rather than being demonstrated at the level of the mediators involved. Polarized-light assessment of collagen fiber organization was not performed either; collagen maturation was instead addressed by direct immunohistochemical typing of collagen I and collagen III, which is a more specific approach to answering this particular question, although it does not describe fiber thickness or spatial arrangement.

In this rat model of frozen shoulder, oral colchicine improved both abduction and external rotation, reduced capsular connective tissue formation and inflammatory change, and lowered capsular collagen III during the inflammatory phase and collagen I during the fibrotic phase, providing concordant functional and histopathological findings. These data show reduced collagen deposition; they do not establish the inhibition of a specific profibrotic pathway. The effects on pannus formation, synovial hyperplasia, and angiogenesis were less consistent and did not reach significance. Colchicine has long been used for rheumatologic disease, and its safety profile in humans is well characterized; however, the present data support further preclinical evaluation rather than the use of colchicine as a conservative treatment for frozen shoulder. Given the modest sample size and the *ex vivo* ROM measurements, these results should be regarded as preliminary, and adequately powered preclinical studies and, ultimately, clinical trials (including a comparison with corticosteroids) are needed before colchicine can be recommended for clinical use. Any such trial would address established adhesive capsulitis, a setting this study did not model.

## Data Availability

The original contributions presented in the study are included in the article; further inquiries can be directed to the corresponding author.
